# A virtual reality task based on animal research – spatial learning and memory in patients after the first episode of schizophrenia

**DOI:** 10.3389/fnbeh.2014.00157

**Published:** 2014-05-27

**Authors:** Iveta Fajnerová, Mabel Rodriguez, David Levčík, Lucie Konrádová, Pavol Mikoláš, Cyril Brom, Aleš Stuchlík, Kamil Vlček, Jiří Horáček

**Affiliations:** ^1^Prague Psychiatric CenterPrague, Czech Republic; ^2^Department of Neurophysiology of Memory, Institute of Physiology, Academy of Sciences of the Czech Republic v.v.i.Prague, Czech Republic; ^3^Department of Psychiatry and Medical Psychology, 3rd Faculty of Medicine, Charles University in PraguePrague, Czech Republic; ^4^Department of Software and Computer Science Education, Faculty of Mathematics and Physics, Charles University in PraguePrague, Czech Republic

**Keywords:** schizophrenia, spatial navigation, learning and memory, virtual reality environment, cognitive deficit, Morris Water Maze (MWM), psychotic disorders, spatial behavior

## Abstract

**Objectives:** Cognitive deficit is considered to be a characteristic feature of schizophrenia disorder. A similar cognitive dysfunction was demonstrated in animal models of schizophrenia. However, the poor comparability of methods used to assess cognition in animals and humans could be responsible for low predictive validity of current animal models. In order to assess spatial abilities in schizophrenia and compare our results with the data obtained in animal models, we designed a virtual analog of the Morris water maze (MWM), the *virtual Four Goals Navigation (vFGN) task.*

**Methods:** Twenty-nine patients after the first psychotic episode with schizophrenia symptoms and a matched group of healthy volunteers performed the *vFGN* task. They were required to find and remember four hidden goal positions in an enclosed virtual arena. The task consisted of two parts. The Reference memory (RM) session with a stable goal position was designed to test spatial learning. The Delayed-matching-to-place (DMP) session presented a modified working memory protocol designed to test the ability to remember a sequence of three hidden goal positions.

**Results:** Data obtained in the RM session show impaired spatial learning in schizophrenia patients compared to the healthy controls in pointing and navigation accuracy. The DMP session showed impaired spatial memory in schizophrenia during the recall of spatial sequence and a similar deficit in spatial bias in the probe trials. The pointing accuracy and the quadrant preference showed higher sensitivity toward the cognitive deficit than the navigation accuracy. Direct navigation to the goal was affected by sex and age of the tested subjects. The age affected spatial performance only in healthy controls.

**Conclusions:** Despite some limitations of the study, our results correspond well with the previous studies in animal models of schizophrenia and support the decline of spatial cognition in schizophrenia, indicating the usefulness of the vFGN task in comparative research.

## Introduction

The impairment of cognitive functions is considered to be a characteristic and permanent manifestation in patients with schizophrenia disorder (Andreasen, 1999; Elvevag and Goldberg, 2000). The MATRICS (Measurement and Treatment Research to Improve Cognition in Schizophrenia) initiative identified seven crucial cognitive areas typically influenced in schizophrenia: attention, psychomotor speed, working memory, logical thinking, problem solving, social cognition, and verbal and visuo-spatial learning (Green et al., 2004). Although the extent of cognitive decline in schizophrenia has considerable inter-individual variability, it has been shown that the overall performance in neuropsychological tests is more than 1 SD lower in schizophrenia when compared to the healthy population (Keefe et al., 2005). This deficit is demonstrated in 82–84% of the patients (Reichenberg et al., 2009).

Various “paper-and-pencil” or simple computer tests are traditionally used to assess cognitive deficit in schizophrenia. However, these methods are not comparable with the behavioral tasks used in animal research and such limitation can be shown in a low predictive validity of the animal models of schizophrenia (Pratt et al., 2012). Considerable attention is therefore devoted to the assessment of visuo-spatial abilities in schizophrenia and in animal models of this disorder, since spatial behavior and spatial memory can be measured using similar methods in various species. It was demonstrated that schizophrenia patients exhibit impaired performance on all levels of spatial cognition, from the most basic level of mental rotations of letters and objects (de Vignemont et al., 2006) to more complex spatial navigation abilities (Weniger and Irle, 2008; Landgraf et al., 2010). Numerous studies demonstrated the deficit of the visuo-spatial working memory in schizophrenia (see review; Piskulic et al., 2007) using various tasks. These findings motivate the development of human analogs of animal spatial tasks for application in comparative clinical research.

**Figure 1:**
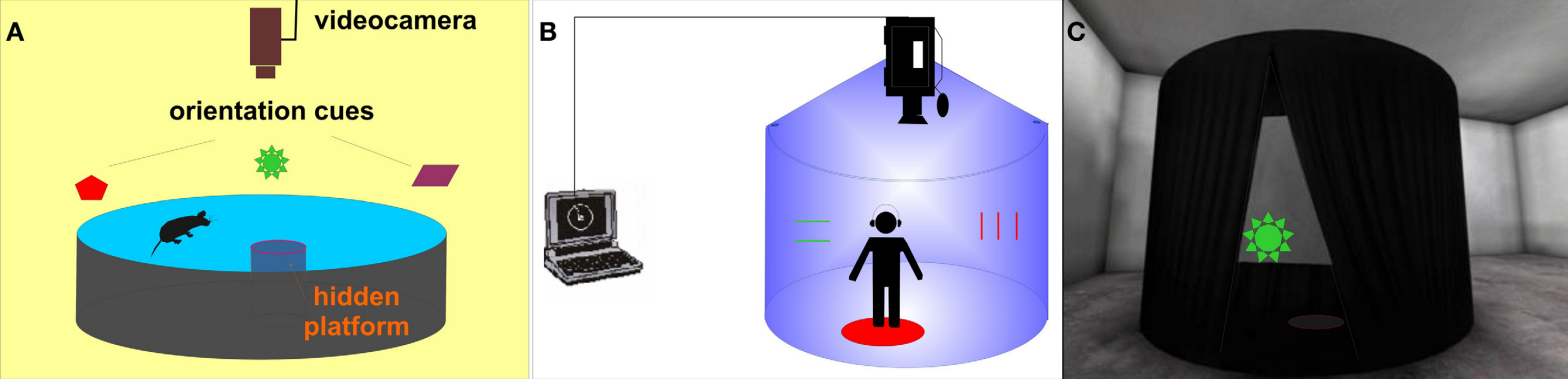
**Morris water maze—the hidden platform paradigm - and its human analogs. (A)** Scheme of the original MWM apparatus for rats. **(B)** Schematic view of the real human analog called Blue Velvet Arena (BVA). **(C)** Virtual analog of BVA (view of the virtual tent from the outside).

One of the most often used spatial tasks in animal research is the *Morris water maze* (MWM; Morris, 1981). This goal-directed task was originally developed for rats and requires them to learn and remember the position of a hidden platform located in a circular swimming pool in relation to distal visual cues (Figure 1A). The MWM apparatus is used in several basic versions (shortly described in Morris, 2008) or protocols (for further information see section Materials and Methods): (1) the *reference memory protocol*, with the hidden platform placed in a stable position; (2) the *reversal protocol*, with a changing platform position; (3) the *delayed-matching-to-place (DMP) protocol* often referred to as the “working memory protocol” which uses variable inter-trial intervals and 4) the *probe trial* with the platform removed. Measurable impairment of visuo-spatial abilities in MWM has already been demonstrated in several animal models of schizophrenia (see review; Bubenikova-Valesova et al., 2008). Several animal studies including the work of our group confirmed that the rodent model of schizophrenia based on administration of MK-801 (dizocilpine, a non-competitive NMDA glutamate receptor antagonist) leads to decreased cognitive functioning in rats, resulting in compromised performance in all variants (reference, reversal, and working memory protocol) of the MWM task (Stuchlik et al., 2004; Vales et al., 2006; van der Staay et al., 2011; Lobellova et al., 2013).

Several real space human MWM analogs have been developed to test the human spatial navigation, mostly in dry circular arenas (Overman et al., 1996; Skolimowska et al., 2011). A real analog of the MWM has also been developed in our laboratory as an apparatus named the “Blue Velvet Arena (BVA)” (Stepankova et al., 1999; Laczo et al., 2010; see Figure 1B). The development of virtual environments (VE) provided a significant methodological advance, allowing the detailed recording of the subject's behavior, along with easy handling and presentation of stimuli. Several virtual reality versions of the MWM have been designed using the reference memory protocol with a stable goal position (Bohbot et al., 1998; Jacobs et al., 1998; Moffat and Resnick, 2002; Astur et al., 2004; Mueller et al., 2008; Goodrich-Hunsaker et al., 2009) or working memory paradigm (Rodriguez, 2010). However, only the reference memory protocol has been applied to schizophrenia patients (Hanlon et al., 2006; Folley et al., 2010).

Thus, our aim was to extend the current comparative research by attempting to incorporate several MWM variants into a small test battery named the “*virtual Four Goals Navigation” (vFGN) task*. The vFGN task is completed in a virtual analog of the real BVA apparatus designed previously by our group (Stepankova et al., 1999; depicted in the Figure 1C). The presented study describes the newly-developed vFGN task and presents first data obtained in a group of patients after the first episode of schizophrenia psychosis in comparison to a group of healthy volunteers, in order to express its sensitivity toward the present cognitive deficit. To minimize possible effects of sex, age and education level, both groups were carefully matched according to these variables. In order to assess the usefulness of the vFGN task in preclinical studies, we compare the data obtained in the vFGN task with the previously published animal studies.

On the basis of animal and human literature, we hypothesized that the schizophrenia patients would perform worse compared to the healthy controls in the vFGN task in terms of: (1) impaired spatial learning during the Reference memory (RM) session; and (2) decreased working memory performance in the Delayed-matching-to-place (DMP) session. Since several studies described sex differences in spatial abilities of rodents (see meta-analysis by Jonasson, 2005) and humans (e.g., Astur et al., 1998, 2004), we hypothesized to find similar differences in our subjects as well. In addition, the effect of age variable was analyzed in order to understand how the age affects performance in the vFGN task and if this effect is the same in both groups. Moreover, the effect of several clinical parameters, such as the duration of untreated psychosis (DUP), general functioning (GAF score), clinical symptoms (PANSS scores) and antipsychotic medication [dose calculated in chlorpromazine (CPZ) equivalents], was evaluated in the group of patients.

## Materials and methods

### Experimental subjects

Twenty-nine patients (17 males and 12 females) after the first psychotic episode with schizophrenia symptoms were recruited for the study. All patients have been diagnosed with schizophrenia or related psychotic disorders according to ICD-10 criteria (Paranoid Schizophrenia F20.0: *n* = 3; Undifferentiated Schizophrenia F20.3: *n* = 1; Simplex Schizophrenia F20.6: *n* = 1; Acute psychotic disorder: F23.0: *n* = 4; F23.1: *n* = 18; F23.2: *n* = 2). They were recruited in the early remission phase during their first psychiatric hospitalization (therefore considered to be first-episode psychotic patients with schizophrenia symptoms, FEP) with a variable duration of untreated psychosis (DUP, 6.4 ± 13 months). DUP defined as the duration of untreated but clearly presented psychotic symptoms, was obtained from the detailed interview with the patients and family members. All of the patients were tested prior to the end of their hospitalization. In order to cover the whole spectrum of the first episodes of schizophrenia, both early and late onset patients were recruited for the study (in the age between 18 and 35 years).

The patients were individually matched to healthy volunteers (*n* = 29; see Table 1) in terms of sex, age (within 2 years difference), education level and gaming experiences (both within 1 level of difference). Healthy subjects were recruited from the same socio-demographic background via a local advertisement. To provide sufficient homogeneity of the examined group, most of the recruited participants were regular users of computer devices with none or mild gaming experience. The inclusion criteria for both groups were: (1) no history of neurological disease or loss of consciousness longer than 10 min; and (2) native in Czech/Slovak language. The main exclusion criterion for the control subjects was personal history of any psychiatric disorder. All tested subjects signed a written informed consent approved by the Ethics Committee.

**Table 1:** **Patients with schizophrenia were individually matched with healthy controls for sex, age (within 2 years), education level and gaming experience (both within one level of difference)**.

	**Group mean (*SD*)**		**Group differences**	
**Demographic variable**	**Schizophrenia patients (SZ)**	**Healthy Controls (HC)**	**Mann-Whitney U**	***p*-value**
N	29	29		
Sex (M: F)	17: 12	17: 12		
Age	25.8 ± 6.2	25.7 ± 5.4	419.5	0.994
Education level (1–6)	3.1 ± 1.6	3.7 ± 1.2	323	0.131
Gaming experience (0–2)	1.1 ± 0.7	0.6 ± 0.5	258	0.012
**Clinical assessment**	**SZ**	**HC**		
PANSS score	56 ± 16	–		
PANSS-positive	13.6 ± 6	–		
PANSS-negative	15 ± 6	–		
PANS-general	27 ± 7.7	–		
GAF	64 ± 20.5	–		
Duration of illness	12 ± 20.8	–		
DUP	6.4 ± 13	–		
Hospitalization duration	30 ± 12	–		
Medication (CPZ equivalents)	426 ± 145	–		
**Neurocognitive assessment**	**Raw test scores–Mean (*SD*)**			
	**SZ**	**HC**	**Mann-Whitney U**	***p*-value**
TMT—*A*	38 ± 12.1	26.5 ± 8	131.5	0.0001
TMT—*B*	98 ± 44	50 ± 11.5	82	0.0001
RCFT—copy	31.6 ± 5	35.7 ± 0.9	98.5	0.0001
RCFT—3 min recall	17.2 ± 8	26.2 ± 5.5	108.510	0.0001
RCFT—30 min recall	17.7 ± 7	26 ± 4.9	4.5	0.0001
Digit Span (WAIS-III)—*forward*	9.3 ± 3.7	10 ± 2.3	254.5	0.09
Digit Span (WAIS-III)—backward	5.3 ± 2.1	7.4 ± 2.1	137.5	0.0001
Spatial Span (WMS-III)—*forward*	8.5 ± 1.8	9 ± 1.4	281.5	0.42
Spatial Span (WMS-III)—*backward*	7.5 ± 2.5	9 ± 1.4	218.5	0.046

SZ, first episodes schizophrenia patients; HC, healthy controls; Education level: 1 = less than high school, 2 = started high school, 3 = completed high school, 4 = started university, 5 = completed university, 6 = started postgraduate studies; Gaming experience: 0 = none, 1 = mild, 2 = good; PANSS, Positive and Negative Symptoms Scale; GAF, Global of Assessment of Functioning; DUP, duration of untreated psychosis; TMT, Trial Making Test; RCFT, Rey-Osterrieth Complex Figure. WMS-III, Wechsler Memory Scale III edition; WAIS-III, Wechsler adult intelligence scale III edition.

### Apparatus and software

**Figure 2:**
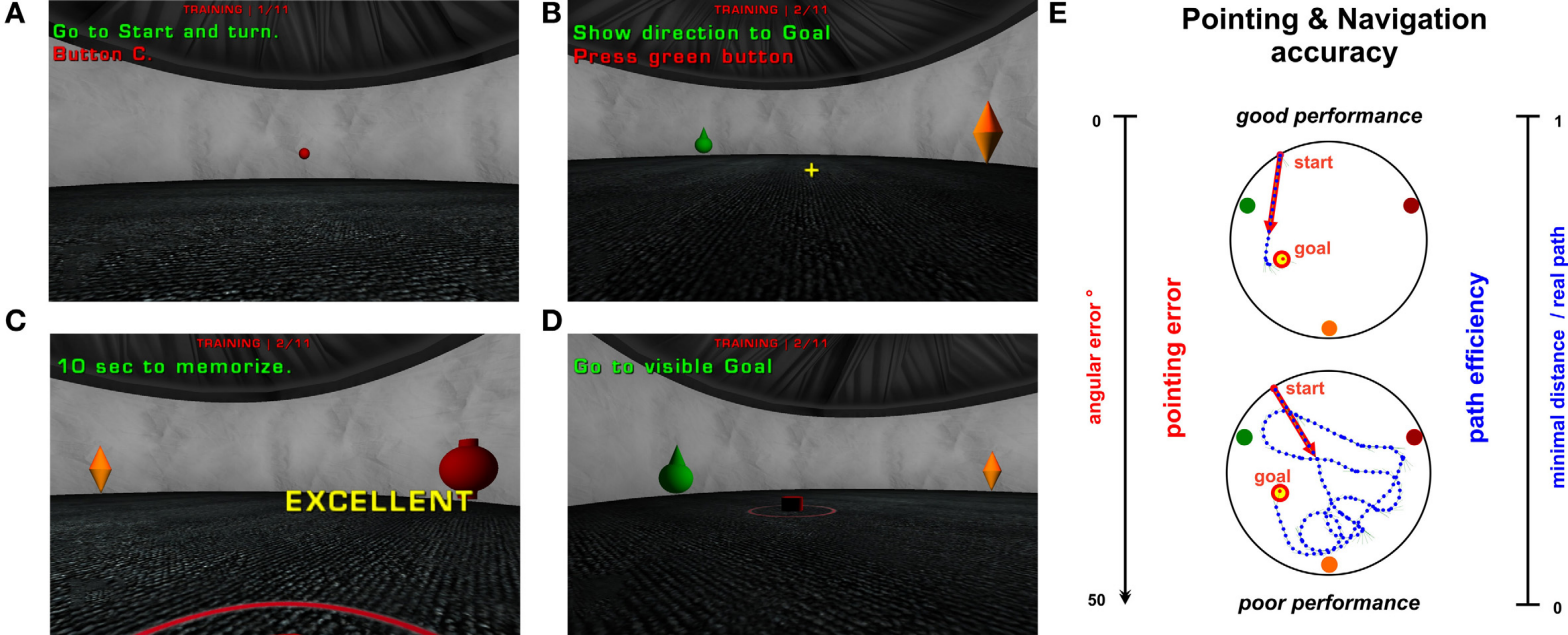
**Virtual version of the BVA apparatus from the inside—one trial procedure. (A–D)** demonstrate individual phases of one trial using the first-person previews from the vFGN task. The short instructions used as a reminder for the participants were translated to English. **(A)** The starting position is presented as a red sphere. **(B)** Two visible orientation cues (from a set of three cues with various shapes and colors) and yellow cross in the middle of the screen used to point toward the hidden goal direction. **(C)** Goal position visualized after 60 s trial time limit. **(D)** Movement blocked after the entrance to the goal position enabling only rotational movement in 10 s memorizing. **(E)** Illustration of the two parameters measured in all trials (except the probe trial). Lateral axis present the interval of values gained in both parameters. The central pictures illustrate good and poor performance in the vFGN task. Trajectories (line of blue points) from the both tested groups are presented as an overview scheme of the spatial configuration in one hidden-goal trial. The red arrow illustrates the pointing error parameter. The trajectory length is transformed to the path efficiency parameter.

The virtual scene was displayed on a 24” LCD monitor using the Unreal Tournament game engine (UT2004; Epic Games, 2004). A Java software toolkit called *“SpaNav”* (Šupalová, 2009) was programmed to configure an experimental setup and to record detailed experimental data for further analysis. A three-dimensional circular arena was designed as a virtual model of the *BVA* apparatus, an arena enclosed by a white curtain wall and with floor covered with a gray carpet (Stepankova et al., 2003), with the utmost realism. Because the virtual environment enabled us to enlarge the size of the virtual arena, an arena 20 times larger than the original BVA apparatus (2.8 m in diameter) was used. Three orientation cues were located in the arena near the circular wall. These objects were fully colored and had various rotational shapes. The goal location had a circular shape with a red border and occupied about 10% of the arena diameter (see Figure 2C). The tested subject moved through the virtual maze in a first person view. In order to facilitate movement in VE for participants without gaming experience, only one stick of the gamepad device (Logitech F310) was used, enabling only forward/backward movement and left/right rotation.

### Clinical and neuropsychological assessment

All of the patients completed a psychiatric interview prior to the experiment in order to obtain information about their current symptoms using the Positive and Negative Symptoms Scale (PANSS; Kay et al., 1987) and the GAF (Global Assessment of Functioning) scale (Jones et al., 1995). Only stabilized patients who mainly scored 3 points or lower in their individual scores were recruited for the experiment. All of the patients were treated by second generation antipsychotics (olanzapin, risperidon, and amisulpirid). The dose of antipsychotic medication was CPZ equivalents (according to Woods, 2003; Andreasen et al., 2010). For details on the clinical parameters see Table 1.

Overall cognitive performance was measured in both patients and healthy controls with several neuropsychological tests compatible with the MATRICS battery to assess psychomotor speed, mental flexibility, learning, and memory (see Table 1): *Trial Making Test* (A and B; Reitan and Wolfson, 1993 modified by Preiss, 1997); *Rey*-*Osterrieth Complex Figure* (Meyers and Meyers, 1995); *Digit span* of the WAIS-III (Wechsler, 1997a); *Spatial span* (computer version adapted from the Corsi block test in the PEBL battery (PEBL, 2012) and modified according to Spatial span of the WMS-III (Wechsler, 1997b).

### Pre-training of motor control

**Figure 3:**
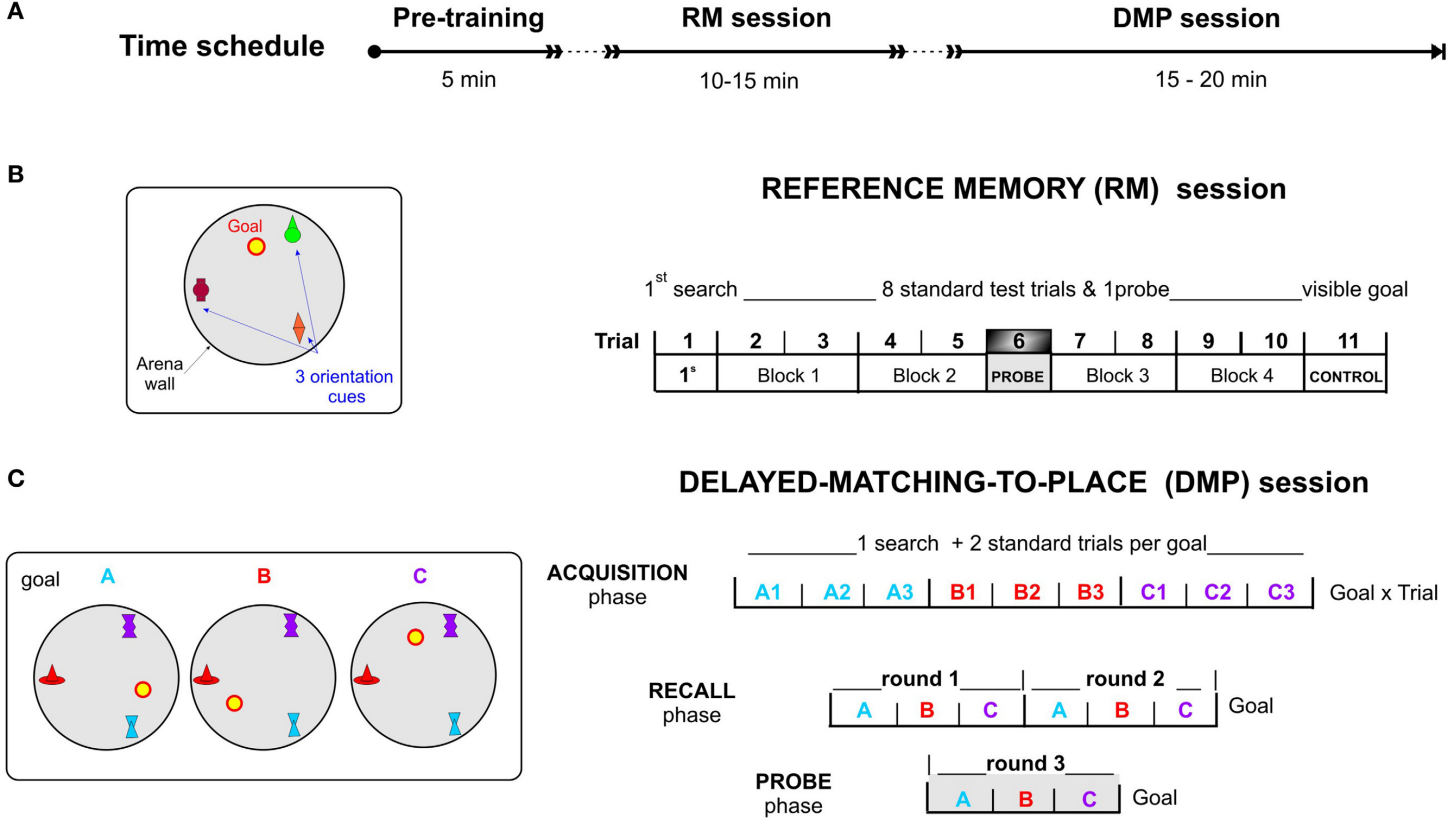
**The virtual Four Goals Navigation (vFGN) task. (A)** upper panel presents the time schedule of the experiment. Spatial configurations with the goal positions used in individual sessions are presented on the left and the procedure of individual parts of the task on the right. **(B)** REFERENCE MEMORY session with a stable goal position over 11 trials. T1, 1st search trial; Block 1-Block 4, pairs of standard trials with repeated search for hidden goal; T6, probe trial with inactivated goal position; T11, control trial testing navigation toward a visible goal. **(C)** DELAYED-MATCHING-TO-PLACE session with goal in three possible positions ordered in a spatial sequence (A,B,C). ACQUISITION phase, each goal position is repeated in 3 consecutive trials (9 trials in total); RECALL phase, two rounds of the spatial sequence ABC (6 trials in total); PROBE phase, one spatial sequence ABC with inactivated goal positions (3 trials).

Prior to the task, all of the participants underwent a short (5 min long) pre-training of movement control using the gamepad apparatus (see time schedule in Figure 3). Afterwards, the participants performed a simple task in a complex virtual labyrinth maze with instructions to “follow the route highlighted by six objects (stars) on each crossroad and get to the end of the route as fast as possible.” After completing the pre-training, all of the tested subjects performed the vFGN task.

### The virtual four goals navigation (vFGN) task

In each trial of the vFGN task the subjects were required to find a hidden circular goal placed on the arena floor using the direct trajectory to the goal. Each trial started by moving toward a pseudorandom starting position displayed as a red sphere near the arena wall (see Figure 2A). Then, three orientation cues were visualized in the arena. At this moment, the subject's movement was blocked at the starting position and only rotational movements were enabled. Apart from the first trial when the goal position was unknown, the subject was instructed to point toward the hidden goal position using the yellow cross in the middle of the screen (see Figure 2B) and then press the green button on the gamepad (in all standard, probe, and control trials) to activate his or her movement. Thereafter, the 60 s time limit for locating the hidden goal began. After entering the correct area, the goal became visible and a short beeping sound was played. If the goal was not found within the 60 s time limit, it became visible (see Figure 2C) and a short warning beep was played. The subject was then instructed to enter the visible goal position. Upon entering the goal area, the movement was blocked in the middle of the goal position and the participant had 10 s to remember the goal position for consecutive trials using only rotational movements (see Figure 2D). This “learning time” represented the analogy of an animal standing on a platform for several seconds after each trial.

The vFGN task consisted of two parts: the RM and the DMP sessions; both administered successively in 1 day protocol (Time schedule in Figure 3).

#### Part I—reference memory (RM) session

**Figure 4:**
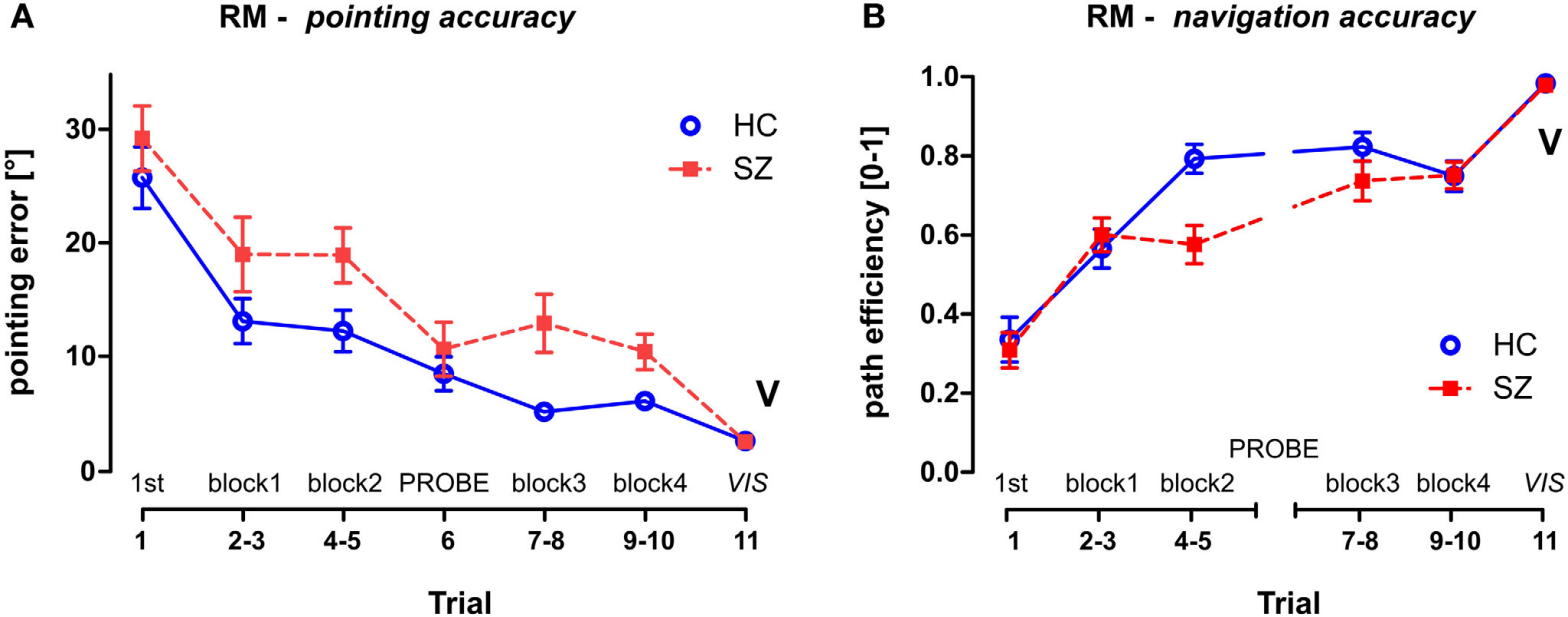
**Reference Memory (RM) session group performance. (A)** The pointing error (mean ± s.e.m) and **(B)** the path efficiency (mean ± s.e.m) measured in individual trials and/or blocks of trials. The probe trial T6 is not depicted in the path efficiency parameter, the control trial T11 is marked by letter V (as displayed). HC—Healthy controls; SZ—first-episode schizophrenia patients.

In the session completed at the beginning of the vFGN task, was designed according to the original reference memory protocol (Morris, 1981, 1984; Morris et al., 1982). Similar to other human MWM analogs (Jacobs et al., 1998; Astur et al., 2002) the task was shortened into 1 day protocol to test spatial learning and memory by monitoring the performance improvement in 11 consecutive trials (see Figure 3B). In the “first search” trial (T1) the participants were instructed to find the hidden goal location on the arena floor by free exploration of the arena and to remember it for the following trials using the three orientation cues. In the following standard trials T2-T5 and T7-T10, displayed as four blocks of 2 trials in Figures 3, 4, the subjects were required to look for the hidden goal repeatedly while starting from pseudo-randomized starting positions. One probe trial (T6) was inserted in the middle of the RM session in order to test the effect of extinction process as a sort of interference in the course of learning (inspired by the human learning tasks). This probe trial was aimed at memory precision and confidence (by evaluating the time spent in the goal proximity) while the goal was inactivated. The final CONTROL trial (T11) used the navigation toward the visible goal and served as a test of secondary effects generated by impairment of vision and motor abilities.

#### Part II—delayed-matching-to-place (DMP) session

In order to prevent any transfer from the RM session, the color and the shape of the orientation cues were changed for the following DMP session. The DMP session was designed as a working memory protocol constructed by combining two different animal protocols for assessment of working memory adapted for humans. The DMP session consists of three consecutive phases (graphically depicted on Figure 3C):

1. The **ACQUISITION** phase involved 9 trials with the goal placed successively in three various positions (A, B, or C) in relation to three distal orientation cues. The goal was moved after each 3 trials (see Figure 3B). It was based on a modified *reversal protocol* of the MWM, in which the goal position is changed over the days used to test mental flexibility (Lipp et al., 1998; Vorhees and Williams, 2006; Garthe et al., 2009; Lobellova et al., 2013) and/or working memory (Morris et al., 1986). Unlike in rats, the change in goal position was separated not by days of testing but by an announcement to the subjects, in order to test their memory for spatial sequence (ABC) in the subsequent phases.
2. The **RECALL** phase together with the Acquisition phase represents a modified version of the DMP paradigm (for review see Dudchenko, 2004; also in Morris et al., 1986; Steele and Morris, 1999; O'Carroll et al., 2006) designed for assessment of the working memory functions in rodents using delayed recall. Our task was designed to test spatial memory processes by evaluating the performance decline measured between the Acquisition and the Recall phase. To increase the difficulty and adapt the task for human participants, the task combined the DMP protocol applied in rats with the spatial sequence encoding in the Corsi Block Test (developed by Corsi in 1972) used in many variants to test spatial working memory in humans (Fischer, 2001). This modified protocol required them to retrieve the correct sequence of three goal positions (ABC) previously learned in the Acquisition trials and identify them successively (according to instructions) in two consecutive rounds (see Figure 3B).
3. The **PROBE** phase, involving 3 trials with inactivated goal position, was conducted directly after the Recall phase as a final third round of the spatial sequenced recall (see Figure 3B). The probe trials, with a removed hidden platform adopted from the animal studies, provide an important demonstration of memory processes in terms of spatial bias (Morris et al., 1982, 1990; Sutherland et al., 1983; Whishaw, 1991). In rats probe trials are usually conducted in the reference memory protocols, but sometimes after reversal condition as well (Lobellova et al., 2013).

### Measured parameters and data analysis

Latency to find the goal and distance traveled to reach it are usually measured in standard trials in animals (for review see D'Hooge and De Deyn, 2001) and in human studies (Hanlon et al., 2006; Moffat, 2009; Folley et al., 2010). In our study the latency parameter was not evaluated, since the decision about the correct goal position was already done while pointing to it. Therefore, we address the spatial performance in all trials except probes using the *pointing accuracy* later referred to as the **pointing error**. This parameter was recorded at the moment when the subject stands on the starting position and points toward the hidden goal by pressing one of the gamepad keys. It was calculated as the absolute angular difference between the pointed and linear direction toward the goal position and its value decreases with growing precision in pointing performance (see Figure 2E). The distance parameter expresses the *navigation accuracy* and it is referred to as **path efficiency** (abbr. **path eff**) with the range of 0–1. It was calculated as a ratio between the minimal possible path length (the actual distance between the start and the goal position) and the real distance traveled by the subject, using the following formula: path eff = path_min_/path_real_ (see Figure 2E). Contrary to the standard *distance* parameter its value increases with the precision of navigation and enables us a direct comparison between individual trials by considering the possible minimal distance. In addition, we measured two common parameters in all of the probe trials: **goal quadrant preference** calculated as a proportion of the overall trial time spent in the goal quadrant (arena quadrant containing the hidden goal in its center); and **number of entrances** calculated as number of crossings through the inactivated goal position.

To analyze the data recorded in SpaNav, a custom-made PHP program called drf2track was used to produce primary data tables and trajectory pictures. Further statistical analysis was performed using the Statistica software (Statistica v.9, StatSoft, Czech Republic). The group differences in the demographic variables (age, education level and gaming experience) are calculated using non-parametric Mann-Whitney U Test. Identical method was used to analyze the raw scores obtained in neuropsychological tests. The group and sex differences in individual parts of the vFGN task were calculated using the GLM repeated measure analysis of variance with two categorical predictors (group × sex). Significant interactions were analyzed using a Newman-Keuls *post-hoc* test. A correlation analysis was performed separately for both groups between the age variable and the spatial performance of individual subjects averaged for individual parts of the vFGN task. The *t*-test for independent groups was used to compare the groups in a single visible goal trial and in a single probe trial in the RM session. The *t*-test for single means against a reference constant was used in order to show that the quadrant preference measured in probe trials is different from the chance level (0.25). The effect of clinical characteristics (age of illness onset, DUP, PANSS, and GAF scores and antipsychotic medication calculated in CPZ equivalents) on averaged performance in the vFGN task was calculated using forward stepwise multiple linear regression analysis (with F to enter set to 1.00 and F to remove to 0). The overall level of significance was set to 0.05.

## Results

**Figure 5:**
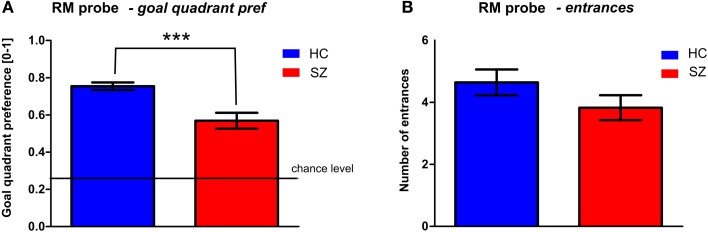
**Probe trial (trial T6) performance in the middle of RM session. (A)** The goal quadrant preference (mean ± s.e.m) and **(B)** the number of entrances (mean ± s.e.m) into the inactivated goal position. Annotation: ^***^p < 0.001 group difference. HC, Healthy controls; SZ, first-episode schizophrenia patients.

**Figure 6:**
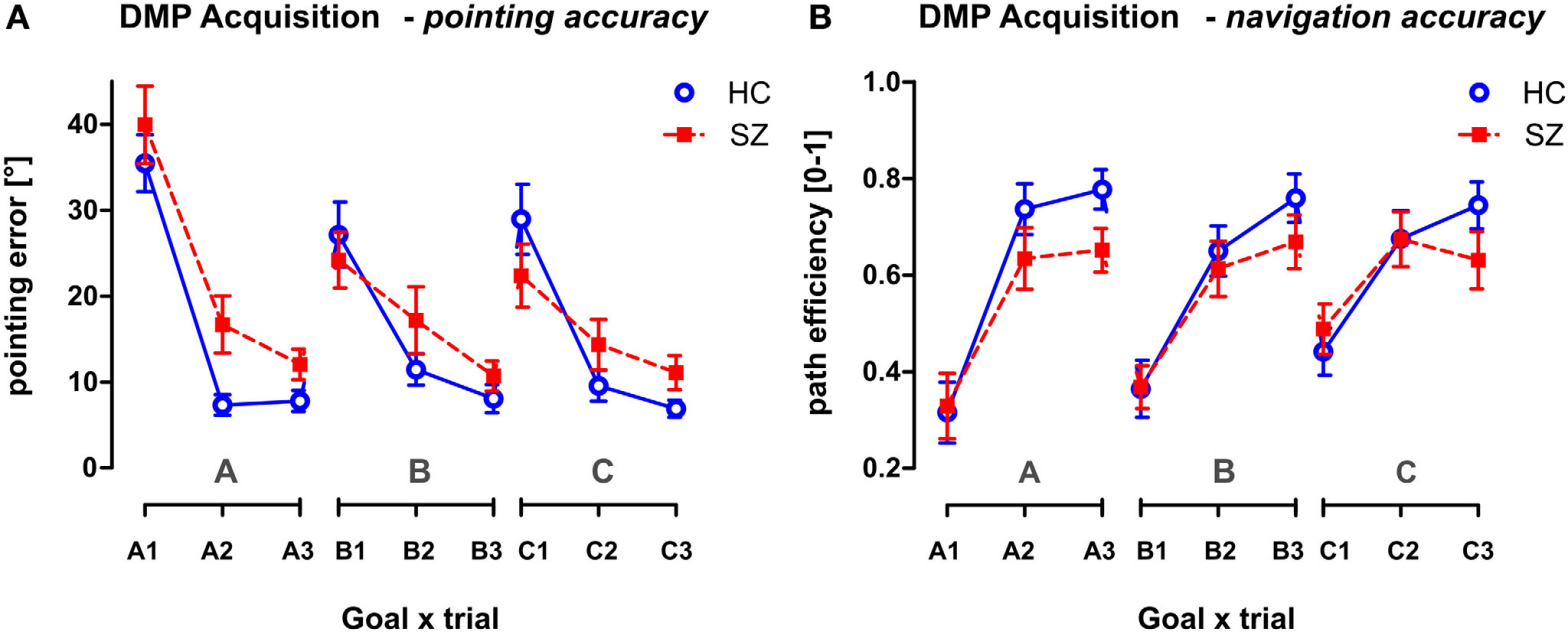
**The performance of both groups in the Acquisition phase of the DMP session**. The behavior is measured after three consecutive spatial changes, the hidden goal is placed in three possible goal positions (A, B, C), in different relationships to the orientation cues. Trials marked as A1, B1, and C1 required the subject to search for the hidden goal after announcement of the positional change. The next 2 trials required repeated search for the hidden goal. The behavior is shown in all 9 trials presented in the order applied during the Acquisition phase using the two following parameters: **(A)** The pointing error (mean ± s.e.m) and **(B)** the path efficiency (mean ± s.e.m). HC, Healthy controls; SZ, first-episode schizophrenia patients.

**Figure 7:**
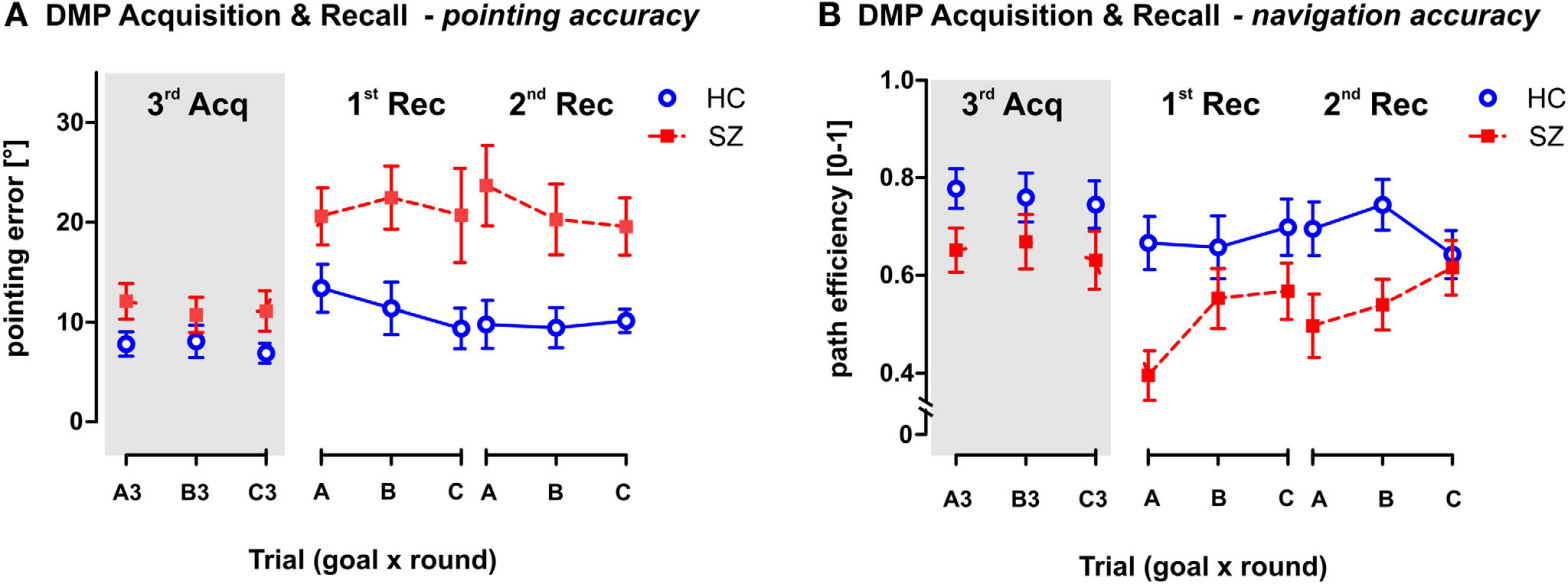
**Pointing and Navigation accuracy in the Recall phase of the DMP session in comparison to the performance in the 3rd trial of the Acquisition phase. (A)** The pointing error (mean ± s.e.m) and **(B)** the path efficiency (mean ± s.e.m) measured in individual trials. Panels 1st Rec and 2nd Rec show the group performance in the two rounds of the Recall phase. Each round requires recalling the previously learned goal positions in the correct sequence (ABC). Gray area on the left (marked as 3rd Acq) represents the performance achieved in the last (3rd) repetitions of each goal position (A3, B3, and C3) in the Acquisition phase. It illustrates the drop in behavioral performance due to time delay between the last Acquisition trial and the Recall phase. HC, Healthy controls; SZ, first-episode schizophrenia patients.

**Figure 8:**
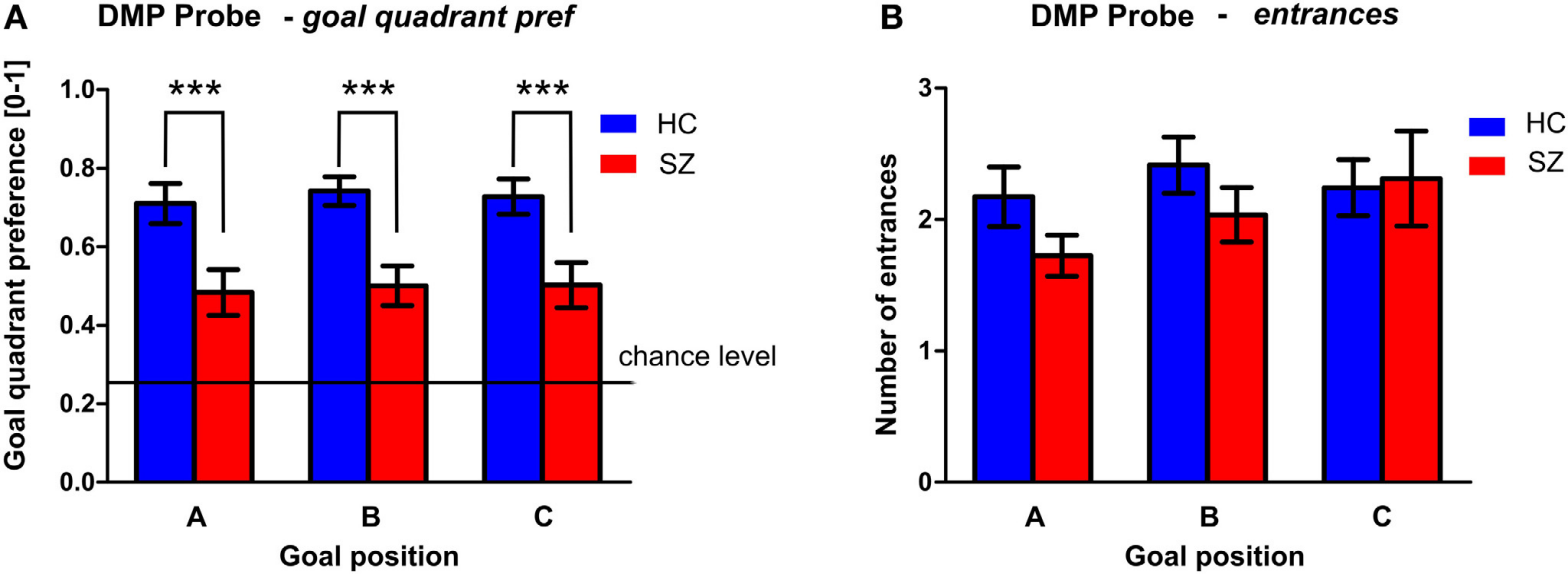
**PROBE phase of the DMP session. (A)** The goal quadrant preference (mean ± s.e.m) and **(B)** the number of entrances (mean ± s.e.m) into the inactivated goal position presented separately for individual goal positions. Annotation: ^***^p < 0.01 group difference. HC, Healthy controls; SZ, first-episode schizophrenia patients.

The groups did not differ significantly in any of the demographic parameters, except the gaming experience, where patients showed to be more experienced than the healthy controls (see Table 1). As expected, group of patients showed significantly lower cognitive performance on all neuropsychological tests, except the forward Digital and Spatial Span task performance (see Table 1). The modification from 3D to 2D version of the Spatial Span could cause lower sensitivity of the test in comparison to other standard methods. Group differences measured in individual parts of the vFGN task are graphically depicted as performance curves for all of the evaluated parameters (see Figures 4–8).

### Group differences in the vFGN task

#### RM session

To analyze the group differences in the RM session a GLM analysis was performed with the group as one of the main factors (group × sex) and block (pairs of standard trials, see Figure 3A) as a repeated measure factor. This analysis showed impaired learning performance of the schizophrenia group in both measured parameters (see Figure 4). While a robust effect of the group factor was identified in the pointing error parameter [*F*_(1, 54)_ = 9.5; *p* < 0.01], a significant interaction (block × group) was found in path efficiency parameter [*F*_(3, 162)_ = 6.2; *p* < 0.001]. A *post-hoc* test on this interaction revealed that the groups differed in path eff (on level *p* < 0.01) in the second block of trials (T4 and T5). Interestingly, the navigation performance in healthy controls improved significantly in the beginning of training between the first two blocks of trials (*p* < 0.001), while in the group of patients similar improvement occurred later on after the completion of probe trial in the middle of the training (only blocks completed before the probe trial showed lower performance than blocks completed after the probe trial; *p* < 0.05). Block as repetition factor was significant in both tested parameters (*p* < 0.001).

To compare the first search trial (T1) of the RM session with the remaining standard trials, another GLM analysis was performed on individual trials. The interaction identified between the group and repetition trials was tested by the *post-hoc* test and revealed that T1 differed significantly from all of the following standard trials in the RM session (*p* < 0.05) in both of the measured parameters. This demonstrates fast learning of the goal position after one learning episode.

One probe trial (T6) was inserted in the middle of the RM session (see Figure 5) to assess spatial memory by evaluating the goal quadrant preference. While the control subjects spent 75 ± 11% of the trial time in the correct arena quadrant, the mean value in the patients was only 57 ± 23%. The goal quadrant preference of both groups differed from the chance level (25%). The *t*-test revealed a main group effect in the goal quadrant preference [*t*_(56)_ = 3.9, *p* < 0.001] but not in the number of entrances to the inactivated goal [*t*_(56)_ = 1.4, *p* = 0.16].

The visible goal trial (T11, marked as V in Figure 4) used as a control of visuo-motor functioning at the end of the RM session showed minimal interpersonal variability. No group effect was revealed by the *t*-test for two independent samples in either of the parameters; in pointing error [*t*_(56)_ = 0.57; *p* = 0.57] or in path eff parameter [*t*_(56)_ = 0.09; *p* = 0.93].

#### DMP—ACQUISITION phase

The main effect of the trial as repeated measures factor was found in the Acquisition phase of the DMP session (*p* < 0.001) tested using GLM analysis (group × sex) with repeated measures (goal × trial) (see Figure 6). The main group effect was found in the pointing accuracy for trials 2 and 3 [*F*_(1, 54)_ = 7.8; *p* < 0.01]. The 1st search trials—A1, B1, C1—representing the free exploration trials were excluded from the analysis as they represent random performance. However, no group differences were identified in the path efficiency parameter, even the interaction effect (trial × group) only approached the significance level [*F*_(2, 108)_ = 2.8, *p* = 0.068]. No other significant interactions were obtained from the analysis.

#### DMP—RECALL phase

The GLM analysis with repeated measures (round × goal) was used to analyze the performance in the two Recall rounds in comparison to the performance observed in the last Acquisition trials—A3, B3, and C3 (see Figure 7). The analysis performed on both Recall rounds showed significant group differences in both measured parameters, as a main effect in pointing error [*F*_(1, 54)_ = 20.4; *p* < 0.001] and in path eff [*F*_(1, 54)_ = 9.9; *p* < 0.01]. Interestingly, while the path efficiency parameter showed only main effect of round as repetition factor (*p* < 0.001), we identified an interaction effect (group × round) in the pointing error [*F*_(2, 108)_ = 4.4; *p* < 0.05]. The *post-hoc* test on this interaction revealed that healthy controls showed stable performance over the DMP session (individual rounds did not differ in the group of healthy controls), but the schizophrenia group showed significant drop of performance after the time delay between the last trials of the Acquisition phase (trials 3) and the first Recall round (*p* < 0.001). No differences have been identified between the two Recall rounds. Interestingly, no main effects or interactions of the goal position were identified.

#### DMP—PROBE phase

The performance of both groups in the PROBE phase (conducted as the last repetition of the spatial sequence after the Recall session) is shown in Figure 8. Despite the fact that the performance of both groups differed from the chance level (0.25), the GLM analysis with goal as repetition factor identified significant group differences in the goal quadrant preference [*F*_(1, 54)_ = 16.9; *p* < 0.001]. However, we found no differences between the individual goal positions. The performance in healthy controls shows that well-trained subjects search for all three goal positions most of the time in the correct quadrant of the arena, as can be seen in their goal quadrant preference of around 73 ± 18%. The averaged performance in the schizophrenia group is lower for all three goals (50 ± 23%). No significant group differences were identified in the number of entrances to the goal position.

### Sex and age differences

In order to show the possible effects of sex on spatial performance in the vFGN task, sex has been used as additional main factor in the GLM analysis (group × sex) with repeated measures performed on individual phases of the task. Interestingly, some sex differences have been observed in almost all parts of the task but exclusively only for the parameter of path efficiency. The main effect of sex was found significant only in the path efficiency in the RM session [*F*_(1, 54)_ = 4.2, *p* < 0.05]. Sex differences approached the significance level in the path efficiency measured in the Acquisition [*F*_(1, 54)_ = 3.6, *p* = 0.064] and the Recall phase [*F*_(1, 54)_ = 3.7, *p* = 0.06] of the DMP session. In all cases males showed superior performance in comparison to females. No differences have been observed either in the pointing accuracy or in the quadrant preference measured in the probe trials. Importantly, no interaction of sex and group factor was observed.

In order to analyze how the age of our participants had affected their performance in the vFGN task, we performed a correlation analysis. The spatial performance of individual participants was averaged for all trials in individual parts of the vFGN task and correlated with the age variable, separately for group of patients and for healthy volunteers (see Table 2). The averaged path efficiency in the group of healthy volunteers negatively correlated with the age of individual subjects in all parts of the task. Similarly, the averaged pointing error in the Recall phase and the number of entrances in the Probe phase was significantly affected by the age variable. Importantly, no such correlation was identified for the group of schizophrenia patients.

**Table 2:** **Correlation analysis between the age variable and the performances averaged for individual parts of the vFGN task, analyzed separately for group of schizophrenia patients and healthy volunteers**.

**Correlation analysis**	**Age correlations**					
	**Schizophrenia patients *N* = 29**			**Healthy Controls *N* = 29**		
**Averaged performance**	***r* (*X,Y*)**	***t***	***p***	***r* (*X,Y*)**	***t***	***p***
avgPath-RM1	−0.48	−2.83	0.009	0.10	0.50	0.62
avgPoint-RM1	0.14	0.77	0.456	−0.20	−1.04	0.31
avgPath-AcqDMP	−0.38	−2.12	0.043	−0.06	−0.31	0.76
avgPoint-AcqDMP	0.33	1.82	0.079	0.10	0.55	0.59
avgPath-RecDMP	−0.49	−2.95	0.006	0.01	0.06	0.95
avgPoint-RecDMP	0.42	2.43	0.022	0.14	0.76	0.45
avgQuadrant- ProbeDMP	−0.36	−2.01	0.054	0.01	0.07	0.95
avgEntrances-ProbeDMP	−0.49	−2.92	0.007	−0.18	−0.96	0.35

avgPath, averaged path efficiency performance; avgPoint, averaged pointing error; avgQuadrant, averaged goal quadrant preference; avgEntrances, averaged number of entrances to the goal; RM1, first half of the RM session; AcqDMP, Acquisition phase of the DMP session; RecDMP, Recall phase of the DMP session; ProbeDMP, Probe phase of the DMP session; r (X,Y), correlation coefficient.

### Regression model of clinical variables effect on performance in the vFGN task

From the set of the potential clinical and demographic factors that could contribute to the cognitive decline observed in the group of patients, the following predictors were added to the regression model (age, DUP, PANSS-P, PANSS-N, PANSS-G, GAF, and CPZ level) analyzing their effect on performance measured in the vFGN task (averaged separately for individual vFGN parts—RM session and three parts of the DMP session). A stepwise forward multiple regression analysis employed using these predictors only identified the following significant effects—positive effect of GAF score on spatial learning ability expressed as the averaged pointing accuracy (b_GAF_ = −0.52; *p* < 0.05) and the navigation accuracy (b_GAF_ = 0.49; *p* < 0.05) in the RM session. The full model was significant only for pointing accuracy (*R* = 0. 57; *R*^2^ = 32%; *p* = 0.045), but not for the path efficiency (*R* = 0. 54; *R*^2^ = 30%; *p* = 0.06), both with GAF and DUP predictors added in 2 steps (all other predictors were removed). In the Recall DMP phase measured by the path efficiency, a significant effect of PANSS-G (b_PANSS−G_ = 0.68; *p* < 0.05) and CPZ level (b_GAF_ = 0.7; *p* < 0.05) was identified. The whole model, with non-significant DUP and PANSS-N as additional predictors added in the succeeding steps, was not significant (*R* = 0.67; *R*^2^ = 44%; *p* = 0.066). No other significant effects were found by applying this regression model.

## Discussion

Both parts of the newly developed virtual vFGN task demonstrated sufficient sensitivity toward the impairment of visuo-spatial functions identified in our schizophrenia patients using standard neuropsychological methods. First, it is important to discuss the sensitivity of the parameters measured in our study. The pointing error parameter has yet not been applied in similar studies, with the exception of the bearing error used to address spatial abilities in a virtual maze (Waller et al., 2001). This pointing error parameter showed higher sensitivity toward behavioral impairment in schizophrenia than the path efficiency parameter. This finding indicates that the simple pointing paradigm could be used to assess spatial abilities separately. Possible explanation of different sensitivity of measured parameters is that the navigation accuracy (expressed in path eff) could be more affected by sex and age differences, connected to skill learning abilities. The common spatial bias parameter (Morris, 1984, 2008) calculated as percentage of time in the correct arena quadrant was more sensitive toward the impairment in schizophrenia than the other applied parameter, the number of entrances to the goal.

### Spatial learning performance in the RM session

The spatial performance measured during the RM session in our participants strengthen the idea of spatial learning impairment in schizophrenia demonstrated in other human studies (Hanlon et al., 2006; Folley et al., 2010) and animal models of schizophrenia (Gorter and de Bruin, 1992; Latysheva and Rayevsky, 2003; Sircar, 2003; Stuchlik et al., 2004). We found decreased performance in the schizophrenia patients in both pointing and navigation accuracy to the goal. However, the navigation accuracy was decreased only in the first half of the RM session.

We were able to demonstrate the continual improvement of performance in healthy controls during the whole RM session, expressed by the decreasing pointing error and path shortening (growing path efficiency). This is in agreement with the evidence that the latency is shortened in animals during consecutive RM sessions (D'Hooge and De Deyn, 2001; Mulder and Pritchett, 2003; Vorhees and Williams, 2006) and in RM blocks tested in human virtual analogs (Nadel et al., 1998; Leplow et al., 2003). Similar continual improvement was present in our group of schizophrenia patients, but interestingly only in the pointing accuracy. In agreement with another human study (Hanlon et al., 2006) the path efficiency of the schizophrenia group did not improve in the first half of the RM session (trial T2-T5). The discrepancy between these two measured parameters supports the idea that navigation performance could be divided into two distinct parts (directional vs. place navigation in Hamilton et al., 2008): (1) selection of direction to the goal at the beginning of the navigation process represented here by the pointing accuracy and (2) precise determination of goal position represented by the path efficiency. We assume that while the patients do improve in directional navigation by remembering the approximate position of the goal (near a particular cue), they do not improve in direct navigation to the goal due to imprecise perception and memorizing of spatial information.

This assumption is supported by the fact that the navigation accuracy improved after the insertion of a single probe trial (in the middle of the RM session) that could facilitate their motivation to focus on important spatial information due to the previous unsuccessful search. This finding supports our assumption that the measured spatial performance is affected by attention deficit measured using standard neuropsychological tests (TMT and Digit span, see Table 1).

In addition, results obtained in the probe trial showed impairment of spatial bias in schizophrenia, in accordance to animal studies (Norris and Foster, 1999; Stuchlik et al., 2004). In rats, the probe test is known to start extinction process; we expected human subjects to respond similarly. The probe trial was therefore applied in the middle of the RM session as a form of interference (often used in learning tasks). Interestingly in animal studies only first half of the probe trial (first 30 s) shows group differences in rodent model of schizophrenia (e.g., Entlerova et al., 2013), as afterwards even the intact animals tend to leave the unrewarded position. However, due to the verbal instruction, our subjects tend to look for the goal during the whole trial. Despite these differences, the human analog of probe trial shows the same pattern as observed in the rodent model of the MWM; lower occupancy of the goal quadrant in the group of schizophrenia patients in comparison to the healthy controls. On the other hand, the number of entrances parameter failed to show significant group differences. This discrepancy indicates that most of the subjects (from both groups) identified the correct goal position, but only the healthy controls tend to stay in the goal area.

Importantly, the final *visible goal trial* showed that the impaired performance observed in the group of schizophrenia patients was not produced by locomotor or sensory deficits. This one-trial finding is in accordance with other human (Hanlon et al., 2006) and animal studies (e.g., Gorter and de Bruin, 1992; Vales et al., 2006), suggesting that the usual block (of several trials) procedure is not essential for demonstrating the control performance of navigation toward a visible goal. Taken together, our findings confirm the designed RM session as a useful tool for assessing visuo-spatial learning in schizophrenia.

### Mental flexibility and working memory performance in the DMP session

#### The ACQUISITION phase

A major performance improvement in the Acquisition phase of the DMP session appeared immediately after the 1st search trial. Similar behavior was also observed in animal studies where only an improvement between the first and the second trial is present in well-trained animals in the DMP or reversal protocol (Garthe et al., 2009; Saab et al., 2011). Despite the observed group differences in the pointing accuracy, the announcement of positional change to our participants was probably responsible for the low group differences in this part of vFGN task. In addition, in order to be able to compare the group performance in later recall of the spatial sequence regardless of individual goal positions, the goals were placed in identical positions (in the meaning of spatial relationship between the goal position and the orientation cues). Such settings could be a source of skill learning effect that could explain the lack of between-group differences observed in the navigation accuracy. Nevertheless, the low sensitivity of the reversal protocol toward the cognitive deficit in schizophrenia is in accordance with the animal studies that failed to find group differences after application of lower doses of MK-801 (Watson and Stanton, 2009; Lobellova et al., 2013). Interestingly, similar reversal protocol applied in the avoidance task on the rotating arena showed that the pre-training of animals in the task (as in our RM session) can lead to lack of group differences after application of MK-801 (Zemanova et al., 2013).

Importantly, the performance of individual groups achieved in the Acquisition phase (in the last repetition of the goal positions A3, B3, and C3) did not differ between the three goal positions, enabling us to test the consecutive recall of this sequence after a time delay.

#### The RECALL phase

Our study was the first to demonstrate impairment in schizophrenia patients using the analog of the DMP MWM protocol. Our results showed impaired recall of spatial sequence in schizophrenia patients in both pointing and navigation accuracy. The working memory performance was here expressed in the performance decrease observed after the time delay between the Acquisition phase and the first round of the Recall phase. The strong performance decline in the group of patients (but not in healthy controls) demonstrates the working and/or long-term memory deficit in schizophrenia. These findings are in agreement with the data obtained in animal models of schizophrenia using the DMP protocol (van der Staay et al., 2011).

#### The PROBE phase

We demonstrated the schizophrenia specific disturbance of spatial bias expressed as decreased *goal quadrant preference* in the PROBE trials completed in the end of the task. The observed behavioral impairment is similar to the observations of rats injected with dizocilpine or scopolamine in pharmacological screening models of schizophrenia and dementia, respectively (Entlerova et al., 2013; Lobellova et al., 2013), which exhibit disturbed performance in probe trials. Nevertheless, in most of the schizophrenia patients the observed probe trial performance was better than in rats after lesion of the hippocampus (Morris et al., 1982; Sutherland et al., 1983) performing by random search patterns.

### Effect of demographic variables

Based on the studies describing sex differences in spatial abilities of both rodents (e.g., Roof and Stein, 1999; Cimadevilla et al., 2000) and humans (e.g., Astur et al., 1998, 2004), we expected to find similar effects in spatial abilities measured by the vFGN task. However, we identified significant sex differences only in learning abilities assessed in the RM session. In addition, sex differences have been observed exclusively for the navigation accuracy (path eff) parameter. This fact and the lack of significant sex differences in other parts of the task suggest the following: (1) the simple circular environment prevents the usage of abilities found to be affected by sex (environmental geometry); (2) the directional information was gained similarly in males and females, yet females tend to use less precise trajectories when navigating toward the goal. This could be due to sex differences in motor skill learning; (3) the animal experiments done with pre-training in MWM protocol showed to exhibit smaller sex differences (Jonasson, 2005), as all the three goal positions have been placed in geometrically identical positions. The lack of interaction between sex and group factor in the measured parameters shows that the presented group effects are independent of the sex differences.

According to the current literature describing negative effect of aging on spatial learning and memory processes (e.g., (Moffat and Resnick, 2002; Moffat, 2009)), we expected to find significant correlation between the age and spatial performance in the vFGN task, both during learning and recall of the spatial information. We confirmed this hypothesis as we observed age effects in all parts of the vFGN task in our healthy volunteers. Interestingly, such effect was fully suppressed in schizophrenia patients. This finding supports the idea that the observed cognitive decline is a characteristic pattern in schizophrenia disorder. This result is in contrary to the current meta-analysis (Rajji et al., 2009), which assumed a better prognosis and less expressed cognitive deficit in patients with a lower age of illness onset. However, our study describes the visuo-spatial deficit only in the early remission phase after the first psychotic episode; repeated assessment in the full remission could reveal a different pattern.

### Effect of psychiatric symptoms and antipsychotic medication

One of the currently monitored clinical parameter is the *DUP* defined as the time from appearance of the first psychotic symptom to the initiation of suitable antipsychotic treatment (for review see; Marshall et al., 2005). In accordance to a recently published follow-up study (Barnes et al., 2008), we found no significant effect of DUP.

Current literature describes a strong association of cognitive functions and negative symptoms, but the absence of a positive symptom effect on cognitive deficit in schizophrenia (e.g., Addington et al., 1991; Rossi et al., 1997). Interestingly, we found no significant effect of negative or positive symptoms on the performance in the vFGN task. However, we observed a strong connection between the GAF score and spatial learning performance in the RM session and effect of generalized symptoms in the Recall phase of the DMP session. These results demonstrate that high-functioning patients perform better in the cognitive tasks than the low-functioning individuals in the group of schizophrenia patients (Green et al., 2004).

Older studies described negative effects of the first-generation antipsychotic treatment on the cognitive functioning in schizophrenia (Spohn and Strauss, 1989). On the contrary, the current studies addressing atypical antipsychotics reported slightly positive effects of some drugs on the cognitive functioning in schizophrenia patients (e.g., Meltzer and McGurk, 1999) and in an NMDA model of schizophrenia in rats (Bubenikova et al., 2005). Interestingly, only the memory deficit found in the Recall phase of the DMP session was affected by the antipsychotic dosage calculated in CPZ equivalents in the navigation accuracy parameter. We did not find any other effect of the atypical antipsychotic treatment on the overall cognitive performance in the vFGN task. Nevertheless, our study was not aimed at individual antipsychotic compounds and this could distort the analysis.

### Limitations of the study

There are some limitations to the current study. Firstly, both animal protocols (RM and DMP) were modified in order to test the human subjects, inducing possible behavioral changes.

The lack of a strong reward motivation present in animal studies (escape from water reaching the platform) could change the motivation to higher performance in the task. However, we assume that our subjects had been motivated as they all voluntarily participated in the study. Moreover, in the group of patients, the vFGN task was performed in the time of neuropsychological assessment aimed to support the diagnostic process. We do believe that during this time period our patients were motivated toward higher performance in general. In addition, both groups judged the level of entertainment during the task similarly as averaged (not reported). Nevertheless, some positive reward could be applied in order to prevent possible lack of motivation in future studies.

In order to enable fast assessment of our participants in only 1 day, the RM protocol could be considered too short to assess long-term memory processes. However, 1-day protocols are common in human studies testing learning abilities and long-term memory in standard tasks (such as verbal or non-verbal learning memory tasks) and in virtual MWMs that have been considered a valid human analogy of spatial RM in rats, and supported by both behavioral data (e.g., Jacobs et al., 1998) and dependence on hippocampal function (e.g., Astur et al., 2002; Goodrich-Hunsaker et al., 2009).

Also the DMP session in our study is not fully comparable to the animal DMP protocol and was modified in the following three details: (1) The inter-trial interval was not controlled directly but was naturally formed by the number of trials included before the recall trial (6 trials for goal A, 4 for B and 2 for C); and (2) Positional changes applied in our study between acquisition and recall of the goal position are not usual in animal studies; (3) The acquisition of the goal position (spatial sequence) was repeated for several (3) trials, as an analog to a reversal protocol, and due to that the spatial information could be retained in the long-term and not the working memory. Despite these modifications we were able to demonstrate that the results obtained in the individual phases of the vFGN task could be compared to the performance patterns obtained in the animal models.

Secondly, despite the smaller number of participants in our study, we were able to demonstrate the deficit in spatial cognition in schizophrenia group. However, matching of the healthy controls to the patients produced an unbalanced distribution in demographic variables, such as the two-peak age distribution in analyzed groups and variable age distribution in males and females caused by the typical age of the early and late onset of schizophrenia.

Thirdly, it is important to note that the navigation performance of schizophrenia patient group observed in the vFGN task was not unitary and showed higher individual variability than the performance in the healthy control group. This variability could be partly produced by the early assessment of patients. Despite these limitations, our findings are supported by the results of studies describing variability of the cognitive deficit level measured in individual first-episode schizophrenia patients (Keefe et al., 2005). In order to understand how the spatial performance was affected by the present attention or memory deficits, further analysis of the spatial performance measured in the vFGN task and its association to standard measures of cognitive deficit is required. A separate paper will be devoted to tracking the possible effects of demographic variables, gaming experiences and cognitive functioning in the group of healthy volunteers, in order to produce normative data for vFGN task performance (in preparation).

## Concluding remarks

The novel vFGN task covered several MWM protocols in a single task and was sensitive toward the impairment of spatial navigation performance, which was observed in nearly all parts of the designed battery. Our results documented strong parallels between the real animal MWM and the presented virtual analog for humans. Therefore, this novel computer task could serve as a useful method of preclinical trials for assessment of spatial behavior and complex cognitive processes in schizophrenia. According to the animal studies, we propose that the vFGN task could be used to assess spatial learning, attention, mental flexibility and spatial working and/or long-term memory processes in three-dimensional space. Future work should confirm the validity of the individual parts of the designed task using a simultaneous examination of the related cognitive functions by standardized neuropsychological methods.

## Future directions

The data presented in this paper demonstrated the sensitivity of the vFGN task toward the cognitive deficit in the first episodes of schizophrenia, confirmed by standard neuropsychological methods. We do believe that the vFGN task assessing complex visuo-spatial behavior could serve as an ecologically valid screening method more sensitive toward the future course of illness in individual patients than the standard methods measuring single cognitive functions. In order to test this sensitivity, a second assessment session takes place 1 year later in the same patients. This time delay is used to evaluate possible cognitive deficit persisting in our patients after the full remission of symptoms or due to potential relapse of the illness. Longitudinal data revealing the trajectory of vFGN performance during the course of schizophrenia are needed.

## Author contributions

Mabel Rodriguez and Iveta Fajnerová designed the study and together with Kamil Vlček wrote the original protocol. Jiří Horáček refined the protocol. Iveta Fajnerová and Kamil Vlček prepared the VR experiment. Iveta Fajnerová, David Levčík and Lucie Konrádová recruited the participants, performed the behavioral and neuropsychological testing and Pavol Mikoláš collected the clinical data. Cyril Brom and his students provided all VR software used in the study. Iveta Fajnerová and Kamil Vlček processed the data and undertook the statistical analysis. Mabel Rodriguez, Kamil Vlček and Jiří Horáček supervised the study. Iveta Fajnerová wrote the first draft of the manuscript. Jiří Horáček, Aleš Stuchlík and Kamil Vlček contributed to data interpretation. All of the authors discussed the results and contributed to the final version of the paper and have approved it.

### Conflict of interest statement

The Review Editor, Malgorzata Julita Wesierska, declares that, despite having collaborated with Aleš Stuchlík, that the review process was handled objectively. The authors declare that the research was conducted in the absence of any commercial or financial relationships that could be construed as a potential conflict of interest.
